# Building the community voice into planning: 25 years of methods development in social audit

**DOI:** 10.1186/1472-6963-11-S2-S1

**Published:** 2011-12-21

**Authors:** Neil Andersson

**Affiliations:** 1 Centro de Investigación de Enfermedades Tropicales (CIET), Universidad Autónoma de Guerrero, Calle Pino, El Roble, Acapulco, Mexico

## Abstract

Health planners and managers make decisions based on their appreciation of causality. Social audits question the assumptions behind this and try to improve quality of available evidence. The method has its origin in the follow-up of Bhopal survivors in the 1980s, where “cluster cohorts” tracked health events over time. In social audit, a representative panel of sentinel sites are the framework to follow the impact of health programmes or reforms. The epidemiological backbone of social audit tackles causality in a calculated way, balancing computational aspects with appreciation of the limits of the science.

Social audits share findings with planners at policy level, health services providers, and users in the household, where final decisions about use of public services rest. Sharing survey results with sample communities and service workers generates a second order of results through structured discussions. Aggregation of these evidence-based community-led solutions across a representative sample provides a rich substrate for decisions. This socialising of evidence for participatory action (SEPA) involves a different skill set but quality control and rigour are still important.

Early social audits addressed settings without accepted sample frames, the fundamentals of reproducible questionnaires, and the logistics of data turnaround. Feedback of results to stakeholders was at CIET insistence – and at CIET expense. Later social audits included strong SEPA components. Recent and current social audits are institutionalising high level research methods in planning, incorporating randomisation and experimental designs in a rigorous approach to causality.

The 25 years have provided a number of lessons. Social audit reduces the arbitrariness of planning decisions, and reduces the wastage of simply allocating resources the way they were in past years. But too much evidence easily exceeds the uptake capacity of decision takers. Political will of governments often did not match those of donors with interest conditioned by political cycles. Some reforms have a longer turnaround than the political cycle; short turnaround interventions can develop momentum. Experience and specialisation made social audit seem more simple than it is. The core of social audit, its mystique, is not easily taught or transferred. Yet teams in Mexico, Nicaragua, Canada, southern Africa, and Pakistan all have more than a decade of experience in social audit, their in-service training supported by a customised Masters programme.

## Epidemiology as a living language between people and public services

Over the last 25 years, several million members of the public and public servants in dozens of countries have participated in CIET social audits of health related issues. Between 1994 and 2010, 45 health sector social audits in 27 countries contacted 504,057 households [1-76]. Additional file 1 summarises the topics, sample and main results.

In the early 1980s, the Italian labour movement “*alternativa operaia*” [77] put forward the idea of community engagement in scientifically defensible epidemiology. Principles like validity of community views, collation of community experience and validation through scientific measurement contrasted the images of white coated scientists coldly observing “subjects” of research. In 1984, follow-up of Bhopal survivors developed sampling and interview approaches that were robust and reliable in developing country conditions. The practical linkages with national health agendas had their roots in UNICEF-sponsored work in Nicaragua and Honduras in the mid 1980s, concerned with evidence on key child and maternal health outcomes [78,79]. At that time, the incompleteness and inaccuracies of routine health record systems in most developing countries all but eliminated the information value of the voluminous but patchy data.

Concerned with the principles behind the Primary Health Care ideal [80] but keen to avoid token community participation [81], the Central American project viewed community engagement as reaching beyond those who used services. For much the same cost as the unreliable routine data collected, in this case on infant and maternal health outcomes across the country, we engaged a sample of communities in a mix of qualitative and quantitative research methods. We tried to optimise information content *and use of evidence* through an inclusive approach that engaged communities and service workers. A cross-design of standard epidemiological and qualitative tools measured common outcomes like diarrhoea and maternal morbidity. We wanted to look upstream from these health outcomes to potential causes – health choices and use of health services.

The idea was not to blend qualitative and quantitative approaches into some half-way method. We broke up the research process or, as in linguistics, we *parsed* it into different moments. Each of these moments had a distinct objective and method. A very participatory moment set the conceptual framework; a more technical moment fitted standard questions to this conceptual framework; in a tightly supervised cluster survey, interviewers read the questions and wrote the answers; an undemocratic data entry moment digitised responses to the questionnaire, with no added value from the keyboard operator; analysis (computation) was technical; a separate community engagement component discussed the results and feasibility of potential solutions, typically through focus groups in each cluster.

The output included multilevel (individual, household and community) data that engaged stakeholders at each level. We worked on two simple principles. First, an epidemiological sample of domains (usually communities) could result in representativeness of the final evidence. Second, repeated cycles of measurement in the same sites could decrease random error of the measurement. Although repeated visits bring other problems, the result was a method to measure health service performance and to understand and to use community engagement in bringing about improvements.

Behind our social audit approach is the idea of epidemiology as an evolving and self-organising system, a language instead of a rigid tool, with increasingly informed community engagement increasing relevance of the emerging solutions. By engaging residents of the clusters or “sentinel sites” in dialogue about their answers to questionnaires, the approach was less about the indicator inferred from a battery of questions following some theory base, and more about what people meant to express and what the enquiry meant to them. At least as important as the first order information about the indicator of health outcome, we found we generated second or third order information of what engaged communities can understand about the indicator and its determinants, what they think can be done about it, and how that should happen. Just as we aggregated vaccination and costs of measles [36], skin conditions [82,83] or seropositivity for Chagas disease [35], we found we could aggregate community-led solutions to those problems into a regional strategy.

## Evidence and guesses in planning

Often defying the risks of reduction and over-interpretation, day-to-day health planning is all about causality. Worse, it is about projections – guesses -- of causality. Planning assumptions are often heroic: the vaccine will be kept and administered correctly; women will attend a prenatal clinic where they will receive what they need; doctors will get it right; medicines will be there and will work. But health services do not always work as expected. They do not reach all those who need them; they do not always have the intended effect for those who use them.

Health services are a live series of subsystems. Health workers have lives to lead, bills to pay, and all this influences health care where it meets the intended beneficiary.

Social audit is a stocktaking of where we are with these assumptions, guesses and intentions. The idea is to produce hard evidence about what works, who is left out and what will make up the shortfall. While a financial audit looks at how financial resources meet financial objectives, a social audit looks at how resources meet defined social objectives. The core activity of stocktaking is to get evidence that tells us about health service performance. The original description in 1985 identified three evidence types -- “words in a common language” [78,79]:

• *Impact* is the change of status (number of diarrhoea cases or a reduction in unofficial payments) attributed to a particular intervention;

• *Coverage* is the proportion who receive a particular service (such as bed-nets, vaccination or access to clean water) out of all those who need it – not only out of those who access the services;

• *Cost* includes time, staff, cash, supplies, transport and all other elements required to supply or to take advantage of a given service or programme. It includes the cost to service users as well as the cost of providing the service.

Linking these three types of evidence in their implicit relationship – coverage of the intervention, that causes the impact, at a given cost – gives meaning to public service performance. This is what most planners want to discuss.

A common failure of health information systems is that evidence comes mostly if not exclusively from institutions. For example, vaccination rates among children who attend a well baby clinic. Or maternal deaths among women who delivered in health facilities. Services that exclude some people by charging too much also exclude these potential beneficiaries from institution-based information systems. One or two percent missing randomly will not affect the big picture much. But some people fail to turn up at health facilities for reasons. If it is those who cannot afford health services, or who have reduced access for reasons of culture or distance, hospital or clinic data will be frankly misleading sources of evidence on the public health. Social audits go to the population base. They find out what people need and what they get, and relate this to the service offer.

The simple fact that social audit goes to the population base opens another dimension. The simpler first order product of social audit is evidence on use of health resources and on service performance. Deliberately engaging the community, or even just “being there”, adds a range of predisposing, enabling and engaging dimensions that affect health and health service behaviour. This is the real science of epidemiology in social audit: understanding, enabling and engaging dimensions and understand how these might affect measurement, and how they might be part of the solution to whatever problem the social audit measures.

## What happens in a social audit

Our 25 years of experience with this approach crystallised a typical sequence of activities in two main phases, summarised in Table 1. We almost always begin with a detailed consultative process, to frame the issues, before reviewing what existing data sources can produce on the problem. The typical sample comes from the latest census, although this is not an invariably reliable sampling frame.

**Table 1:** The two phases of a social audit

Phase 1: design and data collection
•clarify the strategic focus
•analyse existing data to identify gaps and generate operational questions
•design sample, instruments and conduct pilot test
•collect information from households, institutions, and key informants in a panel of representative communities
•link public service and household data, analyse in a way that points to action

Phase 2: socialising evidence for participatory action

•take findings back to the communities for their views about how to improve the situation
•summarise information for policy and management (eg score cards)
•evidence-based training of planners, service-providers and media
•partnerships with civil society

**Figure 1:**
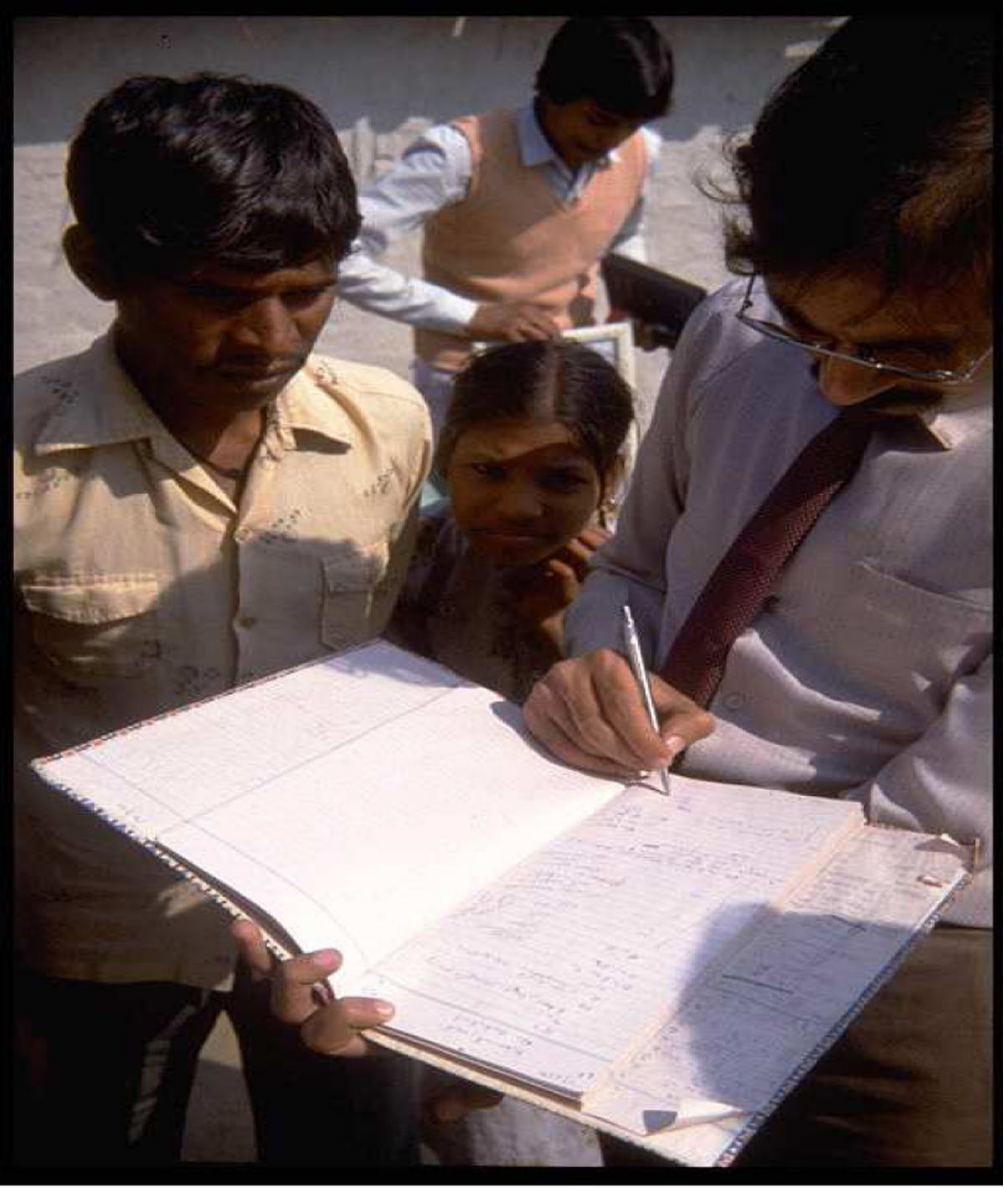
**Bhopal book**. A Bhopal book used during the Bhopal followup. The pages are cut vertically and the questions written on the cover*.*

A household survey usually follows, almost always with face-to-face interviews. The physical data collection instrument commonly associated with our social audits is the “Bhopal book” (Figure 1). We developed it in an emergency setting to collect data from households in a study that achieved 93% five-year follow-up in the aftermath of the infamous Union Carbide disaster in India [84,85]. In the early days simply a school exercise book, this lined ledger has pages cut in half vertically. The interviewer reads the questions from the questionnaire pasted on the inside of the front and back covers and writes the answers on the corresponding line of a page of the book identified for each household, one household per page. Separate pages can serve for different members of a household. The books are inexpensive, usually available locally, robust and reliable in field conditions.

In the household survey, interviewers contact contiguous households in each cluster for statistical handling as a mini universe. This reduces waste of time between households but importantly allows for the interaction between households, for neighbourhood or place, as part of the research process. We link these household data with data from other sources in the same site: institutional reviews of relevant facilities and qualitative data from key informants and focus groups.

Preliminary analysis of this quantitative evidence produces a first round of key findings for discussion in focus groups – in the same cluster -- to gain a qualitative perspective on the findings, particularly views on solutions to the problems. Thus, the household survey permits aggregation of data on occurrence, such as diarrhoea, household opinions of services, costs and so forth. Sharing these results with the clusters, we collect qualitative data on how to deal with these occurrences, in the same clusters. Institutional review of facilities serving the clusters might include local analysis of routinely collected data, personnel issues, times of operation, costs of services and charges, and relations with the community. Some social audits have included a sample of health workers completing a standard questionnaire (like Procol). It might also include observing institutional water supply, curtains for privacy, or even the flow of patients and their treatment.

**Figure 2:**
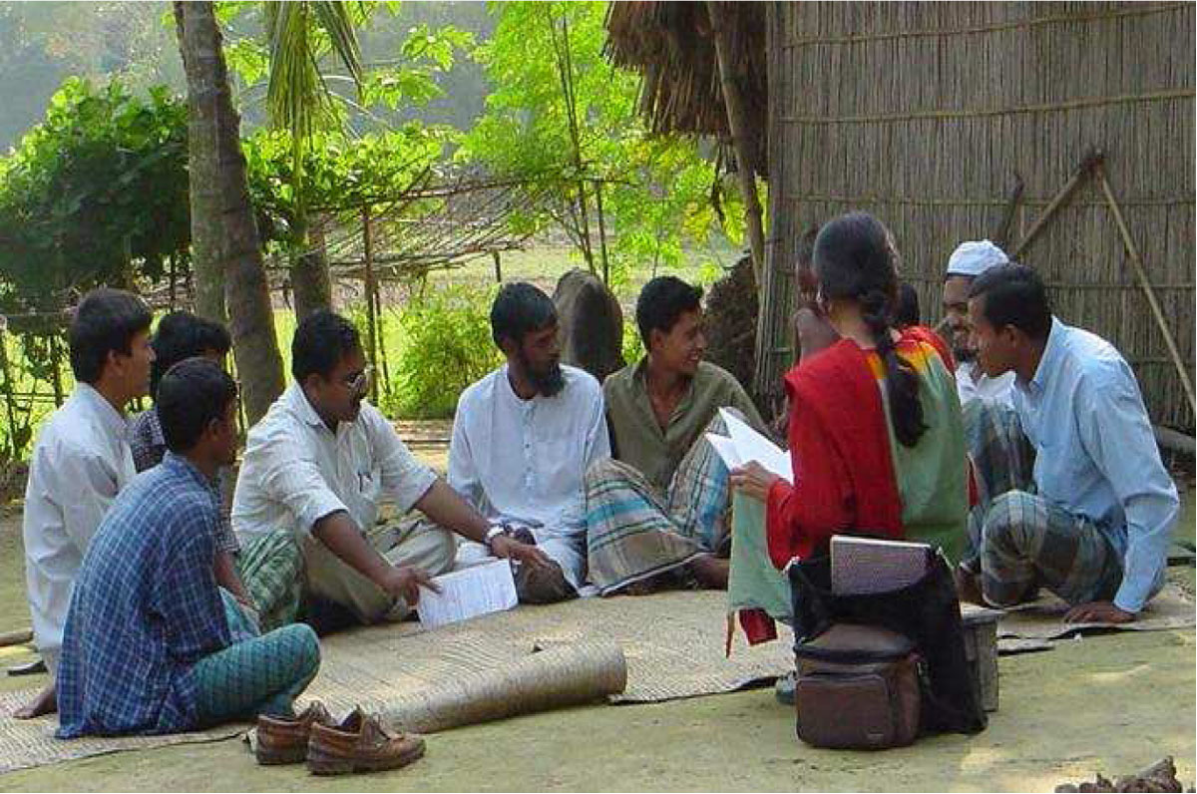
**Focus group**. A focus group discussing access to health care.

The leading epidemiologist analyses the layers of evidence. The research team feeds these preliminary results into discussions of gender stratified focus groups, and with health service workers (Figure 2). Then these results make up the social audit product.

Social audits target three types of research users: planners at policy level, planners in the health services, and planners in the household, where the decisions get taken about health-related actions, including use of public services. Socialising research results involves two feedback dynamics. First, within each sentinel site feedback of findings generates a second order of information – community led solutions. Second, fact-finding and the action it leads to should hold influence beyond the immediate site of data collection^.^ A statistically interpretable sample allows for aggregation of community-led solutions just as it does simple occurrence rates. This allows for assembly of a district or national plan, made up from a representative sample of local plans.

## Methods development and lessons

**Figure 3:**
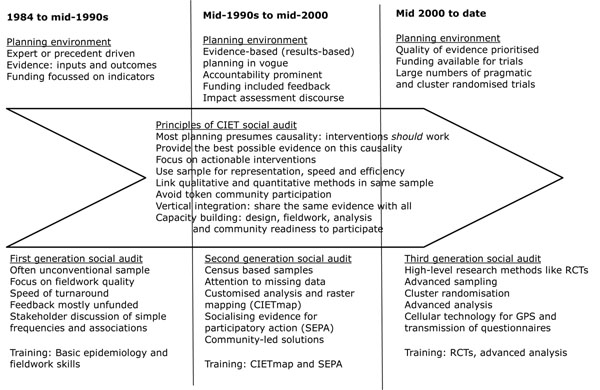
Schematic representation of 25 years of CIET social audits.

Methods developed over the 25 years fall into three generations of social audit reflecting the shifts in demand and supply of evidence for planning. Figure 3 portrays this schematically.

In the mid-1980s in Central America, it was major progress to have reliable evidence on the coverage of key interventions and the indicators of their presumptive outcomes. The first generation of social audit consequently focussed on simple indicators and stakeholder discussions about what could be done about them. Initial challenges included sampling where there was no conventional sampling frame, designing reproducible if not standard questionnaires, and logistics of speedy data turnaround [37]. Analysis focussed on examining associations between coverage and impact, with sequential stratification to deal with potential confounders and modifiers [86,87]. The aim was not to prove causality, but to take discussions one step beyond simple indicators and presumed causality. International organisations funded the surveys as “service delivery surveys” and feedback of findings to spur corrective action was largely at CIET insistence, without funding.

A second development period focussed social audits on methods of feedback and collation of a second order of evidence: what communities and service workers felt could be done about the problems identified in the household surveys. Population weighted raster maps became integral to most social audits, sharing findings with planners who had limited numeracy skills or limited time to absorb findings [4,88].

The third and current generation of social audits incorporates high level research methods to produce data for planning, with a strong focus on analysis methods and, in the area of capacity building, qualifications that could develop careers for trainees [89]. This includes randomised controlled cluster trials in Pakistan [90], southern Africa [91], Mexico [92], and Nicaragua [93].

In reaching this point, we have learnt many lessons: (1) What community-based evidence to get and how often to get it; (2) Combining qualitative and quantitative evidence; (3) Moving social audit results to action; (4) Partner buy-in; (5) Community participation; and (6) Capacity building and human capital for social audits.

### Community-based evidence: what to get, how often to get it, and from where

Social audit *questionnaires* are ideally short, focusing on a small group of related health problems. Participation of counterparts and communities in the design of questionnaires, while desirable, can lead to longer questionnaires as everyone wants to include their own concerns. Institutional reviews are especially prone to collecting information that will never be analysed. It takes dedication and negotiating skills to limit all instruments to items necessary to reach a decision about action. Questioning the use of each item during design sessions is a useful filter.

*Standard questions* and their “validation” are common concerns for those involved in larger scale surveys. Social audits have made use of standards from the earliest days of indirect estimation of infant and child mortality using the standard Brass questions [94]. We have been much less enthusiastic about using batteries of standard questions on culturally dependent issues, like resilience [95]. We use local focus groups and several rounds of piloting to probe the local meaning of questions, and questions with local meaning, during the design stage. This goes further than translation and back-translation, although that happens too.

*Frequency of outcomes*: Usually based on a cluster sample of households, the typical social audit is ideal for common events. It is less useful for rarer events such as cancer or maternal mortality. With maternal mortality the government priority in Nigeria, we used the cluster sample to look at common actionable *risk factors* for maternal mortality, especially gender violence, while a complementary house to house enquiry laid the ground for measuring maternal morbidity [45].

Almost all social audits rest on *voluntary disclosure*, which filters and refracts in unpredictable ways through the gender, education, social class and culture of respondents. Rates of childhood vaccination, unofficial payments and satisfaction with health services all change with type of respondent. Disclosure is a real issue in sensitive topics such as violence against women or extortion by health workers, for fear this might result in withholding services. Almost always, focus groups report higher levels of corruption than do household interviews. Health workers themselves might be cautious about commenting negatively on supervisors, for fear of retribution. In Pakistan [51], we found that simple but standardised measures during training of interviewers can increase disclosure about violence. Interventions to decrease gender violence and corruption will change, often increase, the disclosure rate. This makes it harder to measure impact using a single outcome indicator and underlines the need for careful matching of methods of training and data collection between social audit cycles.

An important problem of a single outcome indicator is that, while services might improve dramatically, this does not always produce a change in the main outcome indicator in the time one can allow between measurement cycles. A partial solution is to use several *intermediate outcomes* and to collate these with the principal outcome. We have summarised these with the acronym CASCADA: **c**onscious knowledge, for example of official costs of services or of danger signs in pregnancy; **a**ttitudes, that it is worth going to prenatal care; **s**ubjective norms, whether most people in the area consider prenatal care worthwhile; the intention to **c**hange, to attend prenatal care for the next pregnancy; **a**gency, the ability to decide to go to prenatal care or to decide where one will deliver a child; **d**iscussion of the options is often a precursor to behaviour change; leading to the **a**ction that can reasonably be expected to have the health impact. We applied this approach in immunisation [62], gender violence [96], and HIV prevention [97].

*Overproduction* of evidence: exceeding the absorptive capacity of government health services was a serious problem in the first years of social audits. As the evidence comes from households that cannot afford to waste their time, this implies a serious lapse. In the Canadian Atlantic provinces, as an extreme example, our contract with Health Canada obliged us to complete two cycles per year in each of four regions; health management systems simply could not respond to this intensity of new evidence. We now use a two year cycle, by the end of which research users are familiar with the evidence, including the community-led solutions.

*Data management* methods have evolved. Our standard practice is double data entry with verification of discordant entries. In several countries we had to convince local statistical bureaux of the need for this by demonstrating their high error rates from their usual single data entry practice. Bhopal books have had an enduring life and in some countries we still rely on these for data collection. Later social audits used scannable self-administered or interviewer administered questionnaires (we used bubbles and Remark software for scanning). We tried several electronic data capture systems over the years. A current social audit in Nigeria uses cellular GPS technology to geo-position the interview, conveying the interview in real time to a distant supervisor.

Another issue is where to get the information – what kind of sample. Sampling lies at the core of methodological rigour and the sample frame changed as CIET social audits evolved from an emergency information aid to an adjunct of peacetime routine health information systems. Where the sample frame was inadequate – and sometimes we had an official sample frame that was simply not credible – we developed listings of all known communities and their estimated size. In one case we used night lights from satellite pictures. In others, we used a purposive sampling method that answered the question “which 30 sites represent the full range of conditions across this region/ country?”

As sampling frames have improved over the last 25 years, we have found the credibility if not the accuracy of the evidence increases with a random sample. Our current standard is multi-stage stratification before last stage random cluster sampling. We stratify the sample into quadrants (regions or provinces) then each quadrant into urban and rural. Random selection from the list in each stratum typically uses probability proportional to population, though we can also oversample sub-populations as required. The cluster sample fits with our need for efficiency and we have optimised our core analytic techniques for this approach. A “transparency table” shows the sample composition next to what is known of the population proportions.

### Combining qualitative and quantitative evidence in analysis

Data management and analysis also evolved. We started off using printed questionnaire sheets to help manual analysis in remote communities using the LT-LW model computer (Large Table-Lots of Work) available in every community back in the 1980s. Adhesive tape divided the surface into a 2x2 table or several 2x2 tables; we counted piles of questionnaires stacked in each cell before manual computation with the aid of a programmable calculator. The arrival of laptops and software like Epi Info changed this, although it limited the analysis in other ways. We went on to develop CIETmap to support our analysis approach and to interface with R, the statistical programming language.

An early challenge of social audit was to include participatory methods [98,99] in an epidemiological framework^.^ In the 1990s, we coined the term meso-analysis to describe the linking of coterminous quantitative and qualitative data on groups of sites – urban/rural, or sites with particular health service characteristics [100,101]. Our preliminary analysis simply took the site level variable into account using stratification. This evolved to include multi-level approaches, not only “taking clustering into account” as leading to overestimated statistical confidence, but allowing that clustering is an important health development dynamic, and trying to quantify its effect [22].

Harvesting qualitative evidence has been another challenge. We conducted thousands of focus groups and have to admit we have simply not exploited the full potential of the emerging evidence. Because we typically do a focus group in each site, and a social audit may deal with hundreds of sites, it takes constant vigilance to stop people counting how many focus groups concluded X compared with how many concluded Y. Several steps can make interpretation of focus group discussions and key informant interviews less mechanical:

Focus groups do not repeat questions in the household questionnaire; they comment on the results, and what can be done about them.

Monitors write down what people say – the words – not counts of how many “agreed”; they note if a view was unanimous or how it was disputed; write content summaries and quotes.

Analysis starts by reading all focus group responses to one topic, with its prompts.

For each topic, monitors try to characterise the issue in words from the group; they report this together with the result used to spur the focus group discussion.

Occasionally, we code a focus group outcome and use it in formal epidemiological analysis as a cluster-level variable; for example, the focus group in some communities might report “bad attitudes” or “language difficulties” of health workers as a possible determinant of uptake of health services.

An advance over the last decade is our incorporation of *cognitive mapping* to engage stakeholders in conceptualising the focus, in design of questionnaires and to systematise indigenous knowledge.

**Figure 4:**
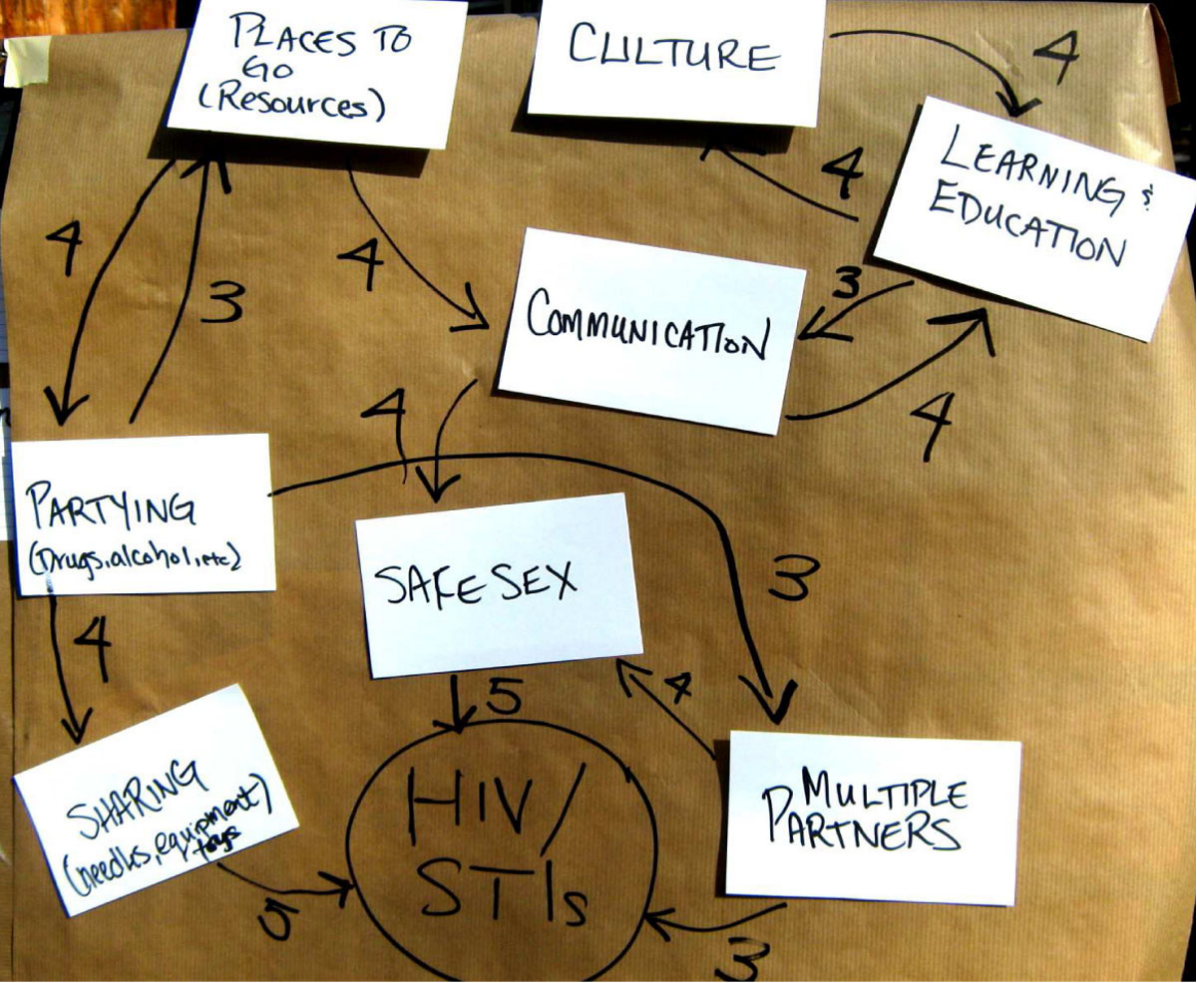
**Fuzzy cognitive mapping**. A cognitive map of prevention of HIV and sexually transmitted infections.

This graphic representation of knowledge of a system or issue comprises concept nodes and causal links weighted according to relative importance (Figure 4). Thus weighted, “fuzzy” cognitive maps (FCM) offer a useful representation of knowledge about causalities that might otherwise seem unstructured and irreconcilable with Western knowledge [102]. Fuzzy cognitive mapping is commonly applied as a group decision support tool to better understand complex factors contributing to a particular outcome or decision [103,104]. We have used FCM to summarise local knowledge and beliefs around a community health issue, contrasting the local belief system around diabetes to that of Western science. This expert knowledge, based on an intimate understanding of the local realities, feeds into various stages of the research process, through formulating hypotheses, questionnaire development, and even data analysis [105].

### Social audit results to action

Evidence is worthless in a report left on a shelf. To have value, our challenge is to translate it into everyday life – usually meaning we must frame it as solutions that people can join into. This does not ignore the expert content of health care, or precision of technologies involved. For example, childhood vaccination involves some very specific technical requirements that do not benefit from participatory action. But people arrange child care or transport to increase their access to vaccination; this is a social and evidence-based process. CIET calls this socialising evidence for participatory action (SEPA).

Effective socialising of evidence at community level requires creativity to compete with the barrage of advertising and the television industry, often with contradictory messages. In Mexico, social audits used song, radio soap operas, community drama, comics and child-to-family schemes [37].

Social audit can help to equip service workers with new tools. In Pakistan, community health workers developed training materials and communication tools using evidence from a national survey on the bond of care between mothers and their children. The health workers embroidered the evidence on a traditional material, so that they could better communicate the concept of risk to mothers [90]. In Afghanistan, focus groups discussed how to discourage people from tampering with landmines (risking death or injury). They concluded that they would trust information about this from the local religious leader or from the BBC world service. Discussions with religious leaders led to inclusion of the issue in Friday sermons. The BBC also included the evidence in their radio soap opera [1,3].

In South Africa, a national youth survey on sexual violence and HIV/AIDS went back to the public through an eight-episode audio-drama that presented the results of the survey and generated discussion that spurred people to think about healthy sexual choices. The audio programme aired on community-based radio shows around the country and curriculum development specialists made it available for life skills education curriculum in schools.

**Figure 5:**
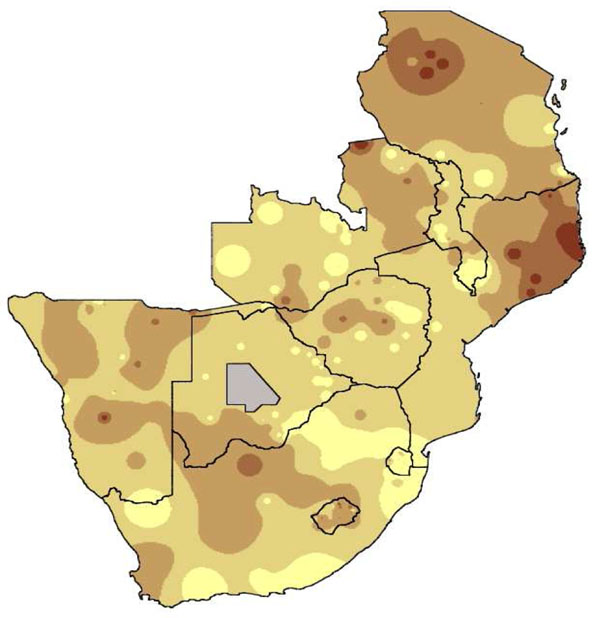
**Raster map**. A population weighted raster map made using CIETmap: % of respondents who do not believe that HIV infected people must live apart from others.

We show elsewhere [106] how population weighted raster maps help to communicate evidence from social audits, especially for non-numerate audiences and settings where broadcasting the average indicator for a sensitive topic is an obstacle to dissemination of evidence (Figure 5). Weighted by the population represented by each cluster/sentinel site, the maps the show proportion of the population affected – adding a spatial dimension to this evidence.

Recent implementation of SEPA in Nigeria began with designation of the state-level priority – in this case, maternal mortality and morbidity. After collation of routine data on first attendances and maternal mortality from every facility in the two states involved in the work, a sentinel process measuring the upstream determinants of maternal mortality reached out to a sample of some 15,000 women in 180 sentinel sites who had been pregnant in the last three years. A household enquiry documented aspects including work in pregnancy, feelings of insecurity, food security, domestic violence and access to care. The field teams discussed results of the household survey (particularly issues of female genital mutilation, domestic violence and work in pregnancy) separately with women and men in every one of the 180 sample clusters. They also discussed findings with health workers and examined health facilities serving each cluster. Analysis tied together this information for feedback and a final layer of data collection – this time about solutions and their feasibility. One page scorecards started discussion on the issue at planning and policy level in each local government authority (district) and at state level. A 15-minute video-drama told the story and raised possible solutions for wider discussion (www.ciet.org/Nigeria/ayihattara). And return visits to the houses of currently pregnant women opened a dialogue about just what it would take to reduce the amount of work they had to do, or what would make them feel safer in their own homes.

This round of data collection focussed on those trying to live the solutions also reduced isolation of women, and it gave a message to men that someone was watching. It changed the ignorance about danger signs in pregnancy and childbirth. Making the same materials available outside the sentinel sites benefits other communities, even if they are not directly involved in the social audit. This increases the impact of the social audit itself, and reduces the differences between sample sites and other places.

### Partner buy-in, or not

A social audit ideally involves government and civil society, from identification of the issues and design of the survey instruments, to analysis of the data and implementation of communication strategies and action plans. This involvement does not always work in favour of quality or detail. In a social audit about childhood malnutrition in one country, the all male steering committee nominated by government blocked the teams asking mothers about their experience of violence before the birth of the child.

A social audit can sometimes be successful while documenting an unsuccessful programme. In South Africa's Eastern Cape Province, a social audit covered five cycles of a regional economic development programme, the Wild Coast Spatial Development Initiative. The development initiative's management ignored community concerns expressed through the social audit, and the undertaking failed [66]. Between 1998 and 2003 the Government of Bangladesh carried out a comprehensive reform of its health services, intended to make them more responsive to public needs. Public opinions and use of government health services fell off during the reform; experience of government services users did not improve [12]. In Bosnia, four linked surveys directed at food sufficiency and vulnerability did not result in a better relief programme or less tracking of food and other resources to the armies [20-22].

Donor enthusiasm for social audit does not necessarily generate government buy in. And without real government buy-in, a technically sound social audit does little for evidence-based planning. In one country, donors pressured the government into a social audit of the targeting of the relief food aid programme, but the government disallowed dissemination of the findings. In another country, government counterparts readily accepted negative findings of the baseline national survey, where they could attribute to this to political predecessors. But official support evaporated after the second national cycle, which showed little improvement and when government counterparts found they could not influence the results. They terminated the contract at the point of socialising the results, and avoided renewing it, despite a further open tender also won by CIET. Initial enthusiasm for the social audit, it turned out, was on the assumption that government agencies would be able to manipulate the results to suit their interests.

A good part of the impact of a social audit lies in the message that it will repeat through hundreds or thousands of questionnaires, scores of focus groups or meetings, and a process of socialising evidence for participatory action. At least one half of the social audits undertaken by CIET have not included a second cycle. In most of these cases, donors precipitated a process that national counterparts did not welcome. Given the limitations of what a single social audit cycle can achieve, especially without allowance for disseminating and using the findings, our current policy is to undertake social audits only if there is provision for at least two cycles of data collection, analysis, and use. As a matter of definition, repeat social audits track changes and measure the impact of reallocation of resources. While a single survey may capture the reality at one time, repeated surveys show trends over time, helping to understand the impact under changing conditions. The data collected over reiterative cycles provides a longitudinal perspective of service delivery, monitoring progress and problems in a way that allows planners and policy makers to adjust their approach or to reallocate resources.

The measurement challenge, of course, is to relate the timing of real change to the social audit cycle. We found it useful to include some fast turnaround outcomes in every social audit, like community knowledge of official pricing systems for health services, which is quite easy to change.

### Community participation

**Figure 6:**
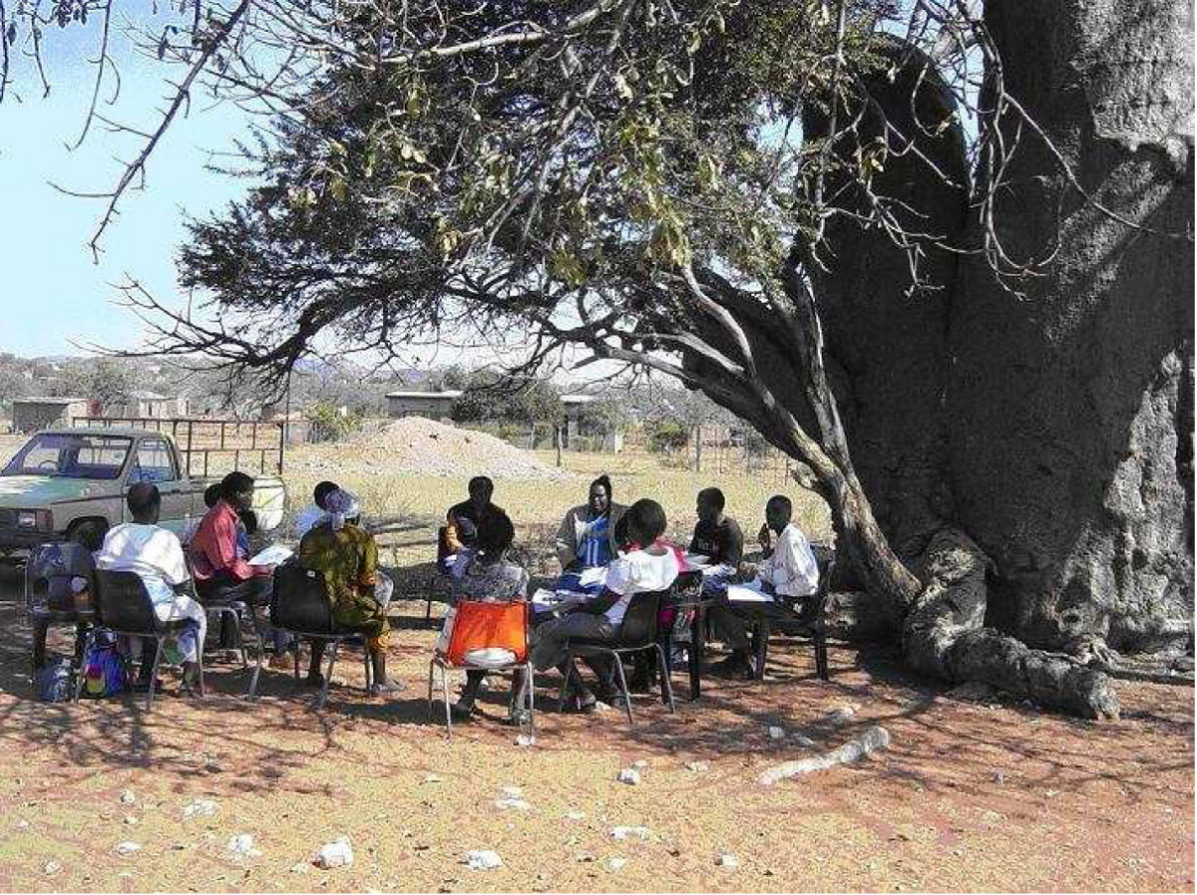
**Focus group, Limpopo tree**. A community discussion group in Limpopo province, South Africa.

When local communities contribute their views through household surveys and discussing the data through focus groups and workshops, social audits become part of a governance network. Active and meaningful participation contributes to empowerment, which implies people’s ability to understand their situation, consider the factors influencing that situation and, most critically, take steps to improve that situation [107] (Figure 6).

Social audit methods raise “collective consciousness” [108]. When communities work together in focus groups or workshops, they talk about their own realities – the information they provided in household surveys. Within the limits of a cross-sectional study, facilitators encourage participants to think about possible causal linkages, and to consider possible actions to deal with likely causes of problems. This can spark individual or collective action to change attitudes and behaviours, to kick off community-initiatives, or to lobby for policy or programmatic shifts.

As with in any health development process, participation of communities in some social audits has been superficial and token, limited to answering questionnaires or sitting through focus groups. The first threshold for meaningful participation is when communities use evidence to generate and to interrogate solutions – what can work and how to make it happen. This is strongly reinforced when they see how their decisions and actions, based on evidence, affect their own health. Even small successes offer big encouragement. A randomised cluster controlled trial in a poor district of Pakistan demonstrated doubling of childhood immunisation rates in sites where groups discussed local social audit evidence about costs and benefits of immunisation and planned what they could do to increase immunisation, compared with control sites [109].

Our approach to community participation has led to a firm division between political protocol, like getting permission of the community leaders to work in the community, and research protocol. This begins with the premise that differences in opinion will exist in every community. There will be differences by gender and generation, by political affiliation and innumerable other divides. We would typically not rely on the community leadership to name or to assemble participants in focus groups, cognitive mapping or action planning. We found it useful to combine focus group recruitment with household interviews. With a definite date and place set for the focus group to discuss the results, the interviewers would each be tasked with getting one particular type of participant. One would invite a woman with children who lives alone for the women's group. Another would invite a woman without children in the home. This we could generate a spread of participants independent of the leadership and most of the social divisions in the community, across a large number of sites. In Nigeria we conducted 180 male and 180 female focus groups; in Pakistan 250 of each, in each survey.

Our approach to community engagement assumes we do not understand, much less that we know how to negotiate, all the tricky layers of local power and patronage. We follow a political protocol for permission to work in the community, and we follow a research protocol to involve as close to a representative spread as we can manage. We do separate consultations for men and women, and sometimes also for younger and older people. We might also have separate consultation processes for particular groups, like women who run male-absent households.

Translation of the results into action, avoiding or at least balancing the interests of the power cliques, requires yet another protocol. The powers that might obstruct or deviate information on a problem almost invariably have a role in changing that problem. The knowledge translation protocol involves sharing the same evidence with at least three constituencies: decision-makers at policy level, decision makers in service delivery and decision makers in the household [110].

Our gender protocols [111] merit particular mention. To begin every social audit, *stratification of existing data by sex* is a useful first step, showing how much was known of the issue “in the system”. This goes beyond disaggregating administrative data by sex. Our second proposition was that it is usually possible to *disaggregate survey data by sex of the respondent and the interviewer.* In most countries, male and female informants give very different quality of information on access to services – especially those relating to children and to gender violence. Third, we argued that engagement around evidence must follow gender lines. *Gender stratified focus groups and cognitive mapping* have proved valuable for conceptualizing problems pre-design, for design and testing of questionnaires, for interpretation of results, and for action planning. The gender concern goes beyond stratification by sex. We separate women into the younger women in the households, in a weak position, and the more senior women in households (the mothers-in- law), in a stronger position. The fourth element involved analysis of *gender related risk and resilience.* For example, household composition is a strong factor for women's access to basic services. The fifth element, to do with the conditions for measuring the associations, is gender appropriate *design and logistic procedures*. This involves women and men, victims and non-victims of gender violence, participating in the design and implementation [112].

Our Social Audit of Abuse Against Women (SAAAW) in Pakistan from 2002-2005 (see Additional file 1) took concern for women's voice to a new level by introducing nuanced training protocols that increased the usefulness of interviewers to participants. Female trainers trained female interviewers to generate a *reverie* just before asking women about their experience of violence. The reverie came from remembering someone the interviewer knew who had suffered abuse, offering a safe space for disclosure by the respondent [51,52] and, as we provided the data for local planning processes, a series of local and regional efforts to decrease violence against women [50][106].

The empowerment implicit in community engagement to generate community led solutions in social audit can have other implications for those who manage resources at state or national levels. At one level, they receive evidence on health issues *and* ideas about what to do to solve these issues. At another level, social audit engages managers or planners with communities in ways that detract from business as usual. This reduces the arbitrariness of whoever pressures most for resources, or the wastage of allocating resources as they were in past years. But not everyone sees this in a positive light. For example, those who benefit disproportionately by being able to pressure, those who benefited from the habitual annual allocations, and those who found they could siphon resources for personal or other objectives, these will not support evidence-based planning. Thus far, we have not been uniformly successful against these interests.

A related concern, where community engagement is successful, is that all this makes the sentinel sites different from the domain they were randomly selected to represent. Repeated cycles can theoretically build social capital, potentially altering the response of the community to a number of health issues. This is the heart of a large RCCT of dengue control in Nicaragua and Mexico, which will compare the specific anti-dengue effects with the general community-mobilising effects. In most settings, the mobilising effect of social audit is not a serious measurement issue. First, the scale of the social audit does not compare with all the other things people have to face in their lives. If the social audit focuses on unofficial payments, it is unlikely to affect other unrelated issues, like diarrhoea rates or knowledge of HIV transmission. A specific aspect might change. Second, the degree to which it changes out of synch with the rest of the domain depends on the SEPA phase. This should unfold the evidence to the entire domain of the sample, not just the sentinel sites, making much of the “special knowledge” available to everyone.

### Capacity building and human capital

Within real funding constraints, all our social audits build capacity of local counterparts. We place particular emphasis on development of epidemiology capacity in non-governmental organizations, local universities and local governments with otherwise few research resources. In the longer term, social audit is best run by a third party, perhaps a civil society organisation or university. Capacity building starts when stakeholders from government and beyond take part in the design process and learn about how to design and test survey instruments, contributing local knowledge content. For data collection, we try to train people from local areas as fieldworkers. This ensures that the researchers are knowledgeable about local language and customs. It also builds capacity in the subject of the survey.

Over the course of four social audit cycles, we found we can build the skills needed to sustain social audit. In Pakistan, three provincial coordinators received academic training in CIET institutions in Canada and Mexico. Government officials from the district and provincial administrations and the Bureau of Statistics have participated in fieldwork and data entry. At least 200 fieldworkers have also been trained to conduct household surveys and to run focus groups. During the workshopping of social audit results, hundreds more government and NGO officials have received exposure to the concept and practical aspects of the social audit. In Nicaragua, the now self-sufficient CIET trained team has run as a social audit enterprise for more than 15 years. Their experience and competence have led them to secure contracts in several neighbouring countries.

Because a social audit is conceptually simple, it is easy to underestimate the need for sustained rigour and quality control. Using a thoroughly tested approach and with technical teams who have together accumulated hundreds of person years of social audit experience, our mistake has been to make the process look too easy. This has led to some donors and national governments adopting the “see one, do one, teach one” approach for national counterparts, often with minimal funding allowance for capacity building. The results have been dreadful, and have done nothing for the standing of social audit as a necessary and useful adjunct to health information systems.

The next phase of social audit will focus on formalising skills and qualifications for the job. This includes a Masters programme with a new distance learning modality, and a doctoral programme, both based at CIET in the Universidad Autónoma de Guerrero, in Mexico. This training addresses skills like design, logistics, quality control, analysis and risk communication. Practical involvement with a committed team remains the best if not only training ground for the single most important capacity in implementing social audit – what they call in Latin America “la mística” -- enthusiasm for a scientific approach. It spreads easily to communities as they see results of the household survey.

## Conclusions

Our particular approach to social audit combines community engagement and modern epidemiology to evaluate causality in public services and, while doing this, the approach helps to build the community voice into planning. Accuracy of decisions that result from the use of epidemiological methods can give meaning and volume to the community voice, increasing confidence of civil society in its participation in governance and thus service reform. This planning of local actions and seeing their benefits is the basis of controlled trials.

## Competing interests

The author declares he has no competing interests.

## Supplementary Material

Additional File 1 **Service delivery surveys and social audits (1994-2010)** A listing of 45 social audits in the health sector in 27 countries, including surveys of over 500,000 households. For each social audit, the table provides the country, year, topic, sample size and sample domain. It also summarises the main results and conclusions of each social audit. The table lists published articles and internet references for each social audit. Click here for file
