# Supplementary material for: Building the community voice into planning: 25 years of methods development in social audit
Source: BMC Health Serv Res. 2011 Dec 21;11(Suppl 2):S1. doi: 10.1186/1472-6963-11-S2-S1 (PMC3397387; doi:10.1186/1472-6963-11-S2-S1)
Supplement: Additional File 1 — Service delivery surveys and social audits (1994-2010) A listing of 45 social audits in the health sector in 27 countries, including surveys of over 500,000 households. For each social audit, the table provides the country, year, topic, sample size and sample domain. It also summarises the main results and conclusions of each social audit. The table lists published articles and internet references for each social audit. [file 1472-6963-11-S2-S1-S1.pdf]

## Service delivery surveys and social audits (1994-2010)

| Country | Year | Topic | Sample size and domain | Results, key findings | References |
|---|---|---|---|---|---|
| Afghanistan | 1994 | Land mines effects | 7500 households in 37 communities: residents, nomads and displaced people | 12% of households were directly affected by mine explosions. Herding was especially dangerous for nomads. One in twenty households had tried to remove mines, increasing the risk of injury fourfold. Focus groups concluded effective communication channels about mines could be religious leaders and the BBC. | 1 |
| Afghanistan | 1997 | Land mines awareness | 9124 households in 86 communities. Focus groups: 200 with men/children, 7 with women | Participation in direct training about mine details *increased* the risk of being involved in a mine accident, with more injuries to upper limbs, suggesting tampering with mines. Combined programmes, including BBC radio programmes, was more beneficial than were individual programmes on their own. | 2,3,4 |
| Afghanistan | 1997 | Multiple indicator survey | 66,596 people in 11,750 households in 75 districts on all sides of the conflict | Data on infectious diseases, immunization, literacy, water and sanitation, nutrition (anthropometry), food security, agricultural production, education, infant mortality and breastfeeding. A quarter of children had acute malnutrition, and half were stunted. Literacy rates and school attendance were low with a very marked gender gap. Less than half of children 12-23 months had measles immunisation. UNICEF used these data in the design of its reconstruction programme. | 5 |
| Afghanistan | 2009 | Demonstration social audit in two districts | 3283 households in two districts: health services provided under contract with province (SM district) or NGO (NGO district). | Service users rated private facilities more positively than government facilities. Government service users were more satisfied in urban facilities, if the household head was not educated, if they had enough food in the last week, and if they waited less than 30 minutes. Many households were unwilling to comment on corruption in health services; 15% in the SM district and 26% in the NGO district reported having been asked for an unofficial payment. Despite a policy of free services, one in seven users paid for treatment in government facilities, and three of four paid for medicine outside the facilities. Focus groups confirmed people knew payments were unofficial; they were afraid to talk about corruption. | 6,7 |
| Angola | 1999 | Land mines awareness | 2157 households, 1166 schoolchildren, 16 teachers, 32 focus groups, in two provinces of Angola | The National Mine Awareness and Mine Accident Prevention Programme (PEPAM) aimed to use schoolchildren as a vector for increasing mine awareness. Children who received the programme had greater mine awareness and were more likely to talk to their families about mines, but were *less* likely to stay out of known mined areas. The PEPAM materials were beyond the reading skills of many of the children. Teachers felt they needed more training to teach mine awareness. They found the guidance materials difficult to use and mainly relied on simple posters. Community campaigns had limited coverage. | 3 |
| Baltic States (Estonia, Latvia, Lithuania) | 2002 | System leakage in the health and licensing sectors | 10,320 households from 30 clusters in each country, 104 health facilities and 90 focus groups in the clusters | Nearly one half of the respondents did not consider unofficial payments to health workers to be corrupt, yet one half (Estonia 43%, Latvia 45%, Lithuania 64%) thought the level of corruption in government health services was high. Very few (Estonia 1%, Latvia 3%, Lithuania 8%) admitted to making unofficial payments in their last contact with the services. A third of respondents said they would report a corrupt health worker. Around 14% of household members across the three countries gave gifts in their last contact with government services. Users who made unofficial payments were less satisfied than those who did not pay with the service they received. The findings served as a baseline for interventions. | 8 |

| Country | Year | Topic | Sample size and domain | Results, key findings | References |
|---|---|---|---|---|---|
| Bangladesh | 1999-2003 | Health and population sector services | Over 25,000 households, 200 health facilities, 200 key informants and nearly 500 focus groups in each of three surveys. Some 2000 health workers in 2000 and 2003 | Between 1999 and 2003, public rating of government health services as "good" fell from 37% to 10%; the proportion using government treatment services fell from 13% to 10%. Unmet need increased from 3% to 9% of households. Health visits to government facilities fell from 17% to 13%, while visits to unqualified practitioners rose from 52% to 60%. Satisfaction with government service providers' behaviour fell from 66% to 56%. Users were more satisfied when waiting time was shorter, prescribed medicines were available, and they received explanations of their condition. Government services retracted despite increased investment; the public preferred unqualified practitioners over government services. The reforms did not help the poorest people. User satisfaction could be increased if government doctors interacted better with patients and if waiting times were shorter. | 9,10,11,12, 13 |
| Bangladesh | 2000 and 2003 | Assessment of impact of Hospital Improvement Initiative in Sylhet Division | In each survey,300 inpatients and 300 outpatients in five hospitals, and 1200 households and 20 focus groups in communities near the hospitals | Compared with 2000, an outpatient in three of the hospitals in 2003 was more likely to be seen within 10 minutes and for at least five minutes by the doctor, but outpatients were less likely to report receiving all the prescribed medicines from the hospital. A 2003 inpatient was more likely to secure admission without an agent. Although patients' satisfaction with several aspects of care improved over time, most changes were not statistically significant. Households in 2003 were more likely to rate the hospitals as good than in 2000. Use of the hospitals generally did not change, except that more used the medical college hospital for inpatient care in 2003. Focus groups confirmed criticisms of services and suggested improvements. | 14,15,16 |
| Bolivia | 1998 | Perception of corruption in public services | 6,850 households, 1600 businesses | Corruption was rampant in the public sector. Households identified the police as most corrupt and in need of reform. One in every for households had to bribe to receive official services, such as obtaining a birth certificate. Many preferred to use intermediaries. Many (69%) had little information on transaction processes. | 17 |
| Bosnia | 1995 | Land mines | 8,975 households in 66 representative communities | Some 3% of households were affected by land mine explosions. Reporting may be viewed as having military importance, causing reticence. Some 28% of the population was displaced. On average, a single land mine explosion killed 0.54 people and injured 1.4. There were active hostilities during the survey; over half of the land mine episodes were related to military activity, mostly affecting soldiers on patrol. Some 2% of households had tried to remove land mines, with a four fold increase in household injury risk. Some hospital staff, aid workers, and individuals refused to participate in the survey. | 1 |
| Bosnia and Herzegovina | 1994-1997 | Food security, the food aid and cash assistance programmes | 30,000 households in four surveys – 6,000-11,000 per survey- in sites across the conflict | Childhood malnutrition, assessed by measurement of MUAC, increased during the conflict, despite the massive food aid programme. A fifth of children were never breastfed; those from vulnerable households were breastfed for shorter times. Children breastfed for less than 4 months were more likely to be malnourished. Despite intended universal coverage from 1994-1996, 15%, 19% , and 31% each year did not receive food. Under-supply reduced from 30% in 1994 to 4% in 1997. Extrapolation suggested 61% of food distributed did not reach households. Targeting of the later cash transfers program was poor, with large inclusion and exclusion errors. Much of the disbursed cash apparently did not reach the intended beneficiaries and could not be accounted for. | 18,19,20,21, 22 |
| Botswana | 2006 | HIV testing and health services use | Household survey of 1536 people in random sample of communities | 92% of government health service users were satisfied with the service, 96% felt they were treated with respect and 90% were comfortable about confidentiality. Almost all would choose a government clinic for treatment of AIDS. Nearly half thought they were at risk of HIV. Half of those who visited a government facility in the last 24 months were offered an HIV test, and nearly half were tested. A few (8%) who were not asked thought they were tested. Most people (79%) had heard of routine HIV testing and 94% were in favour of it. Over half (55%) of the sample had been tested for HIV within the last 12 months. Nearly all (94%) had | 23 |

| Country | Year | Topic | Sample size and domain | Results, key findings | References |
|---|---|---|---|---|---|
| | | | | heard of ART and thought it could help AIDS. Focus groups identified distance from treatment centres and long queues as problems of access to ART. The government has used the findings in planning. | |
| Cambodia | 1994 | Land mines | 6,090 households in 38 communities selected to be representative. Focus groups in sample sites. | Land mine accidents affected 7% of households; 22% of the population had been forced to move because of land mines. Walking to the fields and between villages was the most risky activity. Agricultural production could have increased by 135% if there were no land mines. Some 2% of people had tried to remove land mines, increasing the risk of land mine accidents fourfold. Over half of land mine victims went into debt to seek medical care. Focus groups favoured community meetings to increase mines awareness. | 1 |
| Canada | 1998-2000 | Local public health infrastructure development (LoPHID) | Up to 4 cycles in each of 5 regions, with about 4000 households each; other groups according to the cycle. | *Breastfeeding:* (Labrador) Most respondents supported breastfeeding but practices were sub-optimal. The findings helped develop a programme to support breastfeeding.<br>*Children's dental health:* (Labrador) Dental caries were common and related to diet and hygiene practices. The health board distributed brochures about the findings to parents and schools.<br>*Youth on risk and resilience: (*NFLD*)* Parental and other factors increased resilience.<br>*Perinatal Care:* (NFLD) attitudes were related to low use of pre-natal services.<br>*Heart health:* (NFLD) Information collected about related behaviours fed into prevention programmes<br>*Perinatal care:* (NNS) Low use of prenatal care and risk behaviours (such as smoking)were related to poorer pregnancy outcomes.<br>*Child health Practices*: (NNS) Many children thought they were overweight and were dieting; physical activity was low. Findings informed school health interventions.<br>*Pre-adolescent decision making:* (ENS) Children gave information about risk taking and related factors. The results informed discussions with parents, teachers and youth. | 24,25 |
| Canada | 2001-2003 | Canada PreNatal Nutrition Programme (CPNP) | 2,523 women, students, service providers and elders in 100 First Nations Bands. Focus groups | Trained community based researchers collected data. Half the women had accessed any prenatal service. Women in non-remote communities, with high school graduation, and with supportive partners were more likely to attend prenatal classes. The most vulnerable women were also less likely to access nutrition counselling, food coupons, and cooking classes. A third of women did not know about available prenatal services. Some services were associated with positive outcomes, such as diet and initiation of breastfeeding. Cost-effectiveness of the CPNP was hard to assess based on available data. Supportive Elders were associated with some positive outcomes. The Assembly of First Nations shared the findings with communities. | 26 |
| Canada | 2006 -2010 and ongoing | Sexually transmitted infections (STIs) | 4 Tlicho communities in North West Territories: 1354 respondents in 2006, 1034 in 2010 | This work followed the overall programme on aboriginal youth resilience to HIV/AIDS (ACRA) which began in 1998. The 2006 survey documented knowledge, attitudes and practices related to STIs. Community Action Research Teams (CARTs) from the communities implemented evidence-based interventions. The 2007 survey found a third of people over 26 years old had participated in at least one CART activity. Participants were more likely to talk openly about condoms but less likely to disapprove of multiple partners. The communities are using the findings to refine their CART activities. | 27,28 |
| Costa Rica | 1996 | Human rights in Upala canton | 1300 households including 15% of the population | Some 29% of residents were born in neighbouring Nicaragua, their vulnerability and sometime illegality putting them at risk of human rights abuses. For women in particular, this obstructs access to the labour market and their security of employment. For children, there are issues of access to schools. | 29 |
| Ethiopia | 2001 | Targeting and impact of relief food aid | 3, 628 households in 3 pilot *woredas*; 29 community key | Food security ("enough food in the last week") was 29%, 58%, and 81% in *woredas* A,B and C, and 17%, 8% and 4% of children aged 6-59 months showed acute malnutrition. Food aid programme missed A-64%, B-31% and C-15%. Vulnerable households were more likely to be programme misses, suggesting ineffective | 30 |

| Country | Year | Topic | Sample size and domain | Results, key findings | References |
|---|---|---|---|---|---|
| | | | informants; 29 village committee members; 40 community focus groups | targeting. Few households were satisfied with the amount of food aid received. Community focus groups disagreed about how strongly food aid should target the very needy. This pilot demonstrated the feasibility of a larger social audit. | |
| Maldives | 1999 and 2004 | Reproductive health services | Households: 2,254 in1999, 2,279 in 2004. 1,132 unmarried youth aged 15-24 in 2004 | The contraceptive prevalence rate for modern methods did not change (31% in 1999 and 33% in 2004). The proportion of women attending four or more antenatal visits rose from 65% to 90%, and the proportion taking iron in pregnancy for at least four months rose from 24% to 64%. Youth respondents had important knowledge gaps and some unhelpful attitudes about sex and gender relations. The government and donors used the findings for planning services. | 31,32,33 |
| Mali | 1995 | Social services | 2,399 households, 68 key informants, 18 focus groups of women | Households identified health as the most important public service, followed by education and agriculture. Some 60% of households were willing to pay for improved services. The most frequent complaints about services were absent and poorly performing staff. Few cited corruption as a problem but focus groups noted that people are so accustomed to paying bribes they do not think of this as abnormal. | 34 |
| Mexico | 1986 | Chagas disease | 20 communities in Guerrero province – all households. 4,372 blood samples | A survey of all households measured knowledge. Household searches looked for the carrier insect. Up to 15% of blood samples tested positive for infection with the parasite. Using portable plastic microscopes, field teams showed faeces of infected bugs to community members to explain the disease. In two heavily infected communities, 15% of 145 cardiograms had changes compatible with Chagas' disease. | 35 |
| Mexico | 1990 | Costs of measles | 9,016 households (50,294 people) in 43 representative communities in Guerrero state | After the 1989-90 measles epidemic, a survey in the CIET sentinel sites investigated the costs incurred by the communities and the health services as a consequence of the outbreak, and compared the costs incurred by the community and the health authorities with what it would have cost to vaccinate all the children in the state. The cost of the epidemic to the communities was the equivalent of 27 working days for each case, based on the local cost of labour, substantially more than the cost of sustaining a universal vaccination project over five years. | 36 |
| Mexico | 1992-1995 | Micro-regional planning | 3,000 households in each of four surveys, covering five impoverished municipalities | Diarrhoea rates declined in all five municipalities, and there were several positive intermediate outcomes from the communication strategies – changing knowledge, household practices and uptake of services. There was a strong link between specific contents of the communication package and the changing knowledge or practices. | 37 |
| Mexico | 2000 and 2007 | Corruption in a medical school | Autonomous University of Guerrero, Mexico. 725 medical students in 2000, 436 in 2007 | In 2000, 6% of students had paid unofficially to enter the school; this fell to 1.6% in 2007. Some students admitted paying a bribe to pass an examination: 15% in 2000 and 18% in 2007. In 2007, students were more likely to have bribed a teacher if they were in the fourth year, if they had suffered sexual harassment or political pressure, or if they had been students for five years or more. Students resented the bribes and wanted the school to discipline corrupt teachers. The university administration changed the system of admissions and examinations, based on the findings of the 2000 survey. | 38 |
| Mozambique | 1994-1995 | Social costs of land mines | Over 9,000 households in 65 representative sites | Landmines affected 250 households, causing 83 deaths. Some 10% of households reported loss of agricultural employment because land was in accessible due to mines. Households with a mine victim were 40% more likely to report difficulty in providing food for the family. Focus groups said artisan cooperatives could provide employment for mine victims. The National Planning Commission of Mozambique assisted with the survey. | 1 |

| Country | Year | Topic | Sample size and domain | Results, key findings | References |
|---|---|---|---|---|---|
| Nicaragua | 1998-2009 | Four national surveys on corruption in public services | 6,000 households in each of four surveys | Users' experience of corruption in municipal government, education and health services decreased. The public perception of corruption decreased less quickly than users' experience of it. Progress with traffic police faltered between 2006 and 2009; public perception of police corruption rose in parallel with drivers' experience. Users' experience of corruption in the courts worsened, but public perception of judicial corruption declined. Confidence in the government's anti-corruption struggle grew from 50% to 60% between 2003 and 2009. Public perception of corruption was related to personal experience, the quality of the service, and the perception that municipal government takes community opinion into account and informs people about how it uses public funds. | 39,40 |
| Nicaragua | 1999-2001 | Response to Hurricane Mitch; poverty reduction interventions | Households in representative communities: 10,528 and 6,002 in 1999, 7,799 in 2001<br>Community leaders | In 2001, 45% of households said they were worse off than in the previous year, compared with 37% who said this in 1999. Some 47% of households had a single source of income and for 40% of these that source was unstable. Some 10% of households said illness was their main reason for crisis; 56% did not have enough money to cover health costs. A quarter of households reported an increase in serious arguments; 40% of respondents felt they were subject to violence. A third of households said that natural damage to the community was still not repaired. Field teams took back results to all households. The findings were a key input into government and non-government interventions for poverty reduction. | 41 |
| Nicaragua | 2004-2007 | Evidence based dengue control pilot | 3,956 households in 30 sites in Managua | The 2004 survey gathered entomological, serological and interview data and gave information back to the community through house visits and focus groups to support an informed intervention. Community leaders and dengue *brigadistas* led a communication strategy based on local knowledge and experience in seven of the communities. The cycle in 2005 measured the impact of interventions and refined them with community input. This process refined survey instruments, identified key elements for motivation of community participation, and developed a dengue control "package" that could be implemented elsewhere. This project led to "Camino Verde", a randomised controlled cluster trial initiated at the end of 2009 in Nicaragua and Mexico. | 42 |
| Nigeria | 2006 | Demonstration social audit in health sector | 6,000 households in 24 sites across Bauchi and Cross River states | Cross River: One half of households usually used government health services; 66% of them were satisfied. Dissatisfaction and non-use were attributed to costs and lack of medicines. 68% said corruption was increasing.<br>Bauchi: 76% usually used government health services; 62% were satisfied. Dissatisfaction was related to quality of facilities and cost. 60% said corruption was increasing. | 43,44 |
| Nigeria | 2009-2010 | Maternal outcomes | 25,745 women in 180 sentinel sites in Bauchi and Cross River states | The most consistent and prominent of 28 candidate risk factors for non-fatal maternal morbidity was intimate partner violence (IPV) during pregnancy. Other spouse-related factors included not discussing pregnancy with the spouse and, independently, IPV in the last year. Shortage of food in the last week was a factor as was female genital mutilation. Emerging SEPA (socialising evidence for participatory action) programmes focussed on male responsibility during pregnancy. | 45 |
| Pakistan | 1996-1999 | Community voice in planning | Three cycles across three provinces and one district in the fourth province. In each cycle, some 25,000 households and up to 300 focus groups. | *Gender gap in primary education:* The gender gap, especially in rural areas, was less if mothers were educated and had more say in education decisions. Findings led to efforts to encourage men to educate daughters, to recruit female teachers, to strengthen PTAs, and to provide drinking water and girls' toilets in schools.<br>*Water and sanitation risks:* Childhood diarrhoea and poor growth were linked to unhygienic disposal of garbage, lack of access to safe water, not treating drinking water, and not using safe latrines. Communities | 46,47,48 |

| Country | Year | Topic | Sample size and domain | Results, key findings | References |
|---|---|---|---|---|---|
| | | | | suggested the best way to communicate messages and government bodies developed evidence-based action plans. *Bond of care:* Children were more likely to be malnourished if their mothers were less educated, if they had experienced domestic violence, and if they had not attended antenatal care. Findings led to action plans around the identified problems. | |
| Pakistan | 2001-2002 | Access to justice for the women of Karachi | 1,881 women, 38 police officers, 19 police stations, 20 female and 20 male focus groups | Half the women thought they had sufficient access to justice. Few knew of sources of legal assistance, or how the legal system operated. They rarely used the police and courts; they sought justice through family elders. Factors hindering access to formal justice structures included: corruption, fear of stigmatisation, lack of trust in the police and courts, gender differences in information sharing, and loopholes in the laws. | 49 |
| Pakistan | 2001-2004 | Social audit of abuse against women | Household survey of 23,430 women, 9,000 senior women, 1,500 men. 194 focus groups of eligible women, 187 focus groups of men, and 176 focus groups of senior women. | Extensive piloting developed methods for getting disclosure of abuse by women in a household interview. One in three women had experienced physical violence. Only 35% of them had told anyone, nearly always someone within the family. Women who had discussed violence and felt empowered to talk about it were more likely to have told someone about their experience. Of 7,895 women who had suffered physical violence, only 14 had reported it to the police. Female focus groups said women who report violence bring dishonour to the family; reporting violence may increase it or lead to separation and loss of their children. Focus groups were sceptical about secular and and religious leaders supporting reporting of violence. They suggested setting up local groups where abused women could seek help and advice. Government and non-government bodies discussed the findings and initiated actions, including local committees for reporting. | 50,51,52 |
| Pakistan | 2001-2004 | Social audit of governance and delivery of public services | 57,321 households in 2002, and 53,960 in 2004. 800 focus groups for each survey. In 2004, interviews with 1200 government key informants at or below district level | The surveys covered key services, including health services, following devolution of government powers to districts. Few households were satisfied with government health services (23% in 2002 and 27% in 2004). Fewer households used government health services in 2004 (24%) than in 2002 (29%). More users of private services (87%) and private unqualified practitioners (78%) were satisfied. Government service users were more satisfied if they received all medicines or an explanation of their condition. Focus groups said people avoid government health services because of bad treatment from staff, unavailable or poor quality medicines, bad quality of care, and having to pay bribes. District *nazims* and administrators cited problems with implementation of devolution, especially with transfer of funds. Materials developed to disseminate findings included a series of video-dramas. | 53,54,55 |
| Pakistan | 2001-2004 | Social audit in focus districts: preventive child health | 2-3,000 households in each of 5 focus districts; review of facilities and focus groups in each site. | Devolved district governments in the 5 districts requested help with collecting and using local evidence to improve district-level planning, and agreed a priority topic of preventive child health. District counterparts participated in design, implementation and interpretation of findings. The findings highlighted considerable differences between districts in prevalence of problems and associated factors. Each district developed action plans, with technical support, based on their own specific evidence. Lack of funds transferred from central government thwarted many of the plans. | 56,57,58,59,60,61,62 |
| South Africa | 1997-2000 | Sexual violence in South Johannesburg | 3 surveys in 38 sites in South Johannesburg, each including: women, men, school children; police & justice services; focus groups | The surveys identified unhelpful attitudes among men, women and school going youth: violence against women is acceptable, women mean Yes when they say No, women are partly responsible for sexual violence, women enjoy being raped. Many women and girls held views similar to those of men and boys. A fifth of men said they had forced a woman to have sex. Half of women said a mother might allow abuse of her daughter because of economic necessity. By age 18, 20% of girls and 13% of boys reported they had been sexually abused. Few rapes were reported to the police and very few ever came to trial. As a result of the work, the | 63 |

| Country | Year | Topic | Sample size and domain | Results, key findings | References |
|---|---|---|---|---|---|
| | | | | police changed some procedures and a number of community based initiatives got underway. | |
| South Africa | 1997-2007 | Local economic development in the Wild Coast | Some 24,000 households in 1997, 2000, 2004 and 2007. Small business reviews, key informants, 40 male and female focus groups each cycle. | Awareness of the Wild Coast Spatial Development Initiative (SDI) fell from up to 40% to zero. In 2007 only a quarter of households could name any development project. The proportion getting water from a protected source rose from 20% in 1997 to more than 50% in 2007; while the proportion producing food fell (1997 -71%, 2004 -21%, 2007 -32%). Most households used government health services; the proportion paying for service fell from 26% in 2000, to 7% in 2004, to 2% in 2007. Some 7% owned a small business in 2007, unchanged since 1997. Less households tried to get a loan in 2007 (17%) than in 2000 (42%); almost none were for starting a business. Far more used a loan shark in 2007 (56%) than in 2004 (35%) or 2000 (2%). | 64,65,66,67, 68 |
| South Africa | 2001 | Social sector service delivery standards | 2,297 households in Amatole district, Eastern Cape Province | Assessment against *Batho Pele* (People First) standards in the social sector, including health services. A third of households were dissatisfied with services,calling for more medicines in health facilities. While 92% of children were vaccinated against measles, 25% of households did not know that vaccination for children was free. Over half did not know how to complain about health services. Some service users paid unofficial fees. | 69 |
| South Africa | 2001 | Social sector service delivery standards | 1,305 households in two districts of then Northern Province (now Limpopo) | This social audit assessed services against the Batho Pele standards. One in five households was dissatisfied with services. Some 90% of children were vaccinated against measles and almost everyone felt the clinic nurses were accessible to them. Some households, especially poorer ones, did not know that childhood vaccination was free. Two thirds did not know how to make a complaint about health services. More than a third paid for supposedly free health services. | 70 |
| South Africa | 2003-2004 | Social audit of health services | Gauteng province. 5,490 households, 120 focus groups. Health facility reviews, health worker interviews. | The social audit assessed health services against Batho Pele standards. There was little consultation with service users. Some 56% of households were satisfied with health services. Many people were dissatisfied with the way they were treated by health workers. Only a quarter had information about free services. Two thirds did not know how to make a complaint about services. Recommendations based on the findings were taken up by the provincial government. | 71 |
| South Africa | 2004 and 2007 | Access to antiretroviral therapy | Free State province 2004: 4,444 household respondents, 96 focus groups, health workers in 67 facilities | In 2004, only half of respondents had heard of ART. Many had inaccurate information about when to take ART. Only a third of all respondents believed ART can help someone with AIDS. One in six believed condoms cause AIDS. Many focus groups mentioned misconceptions about causes of AIDS. Between 2004 and 2007, CIET implemented three different communication strategies about ART (alone or in combination) in randomly allocated communities. In 2007 most people in all the communities accepted ART.The most effective intervention was the combination of the three strategies; communities with individual strategies were little different from the control communities. In the combined intervention communities, more people had heard of ART and knew the appropriate timing of its use; more believed ART can help someone with AIDS and said they would take ARTs if they needed them in future. | 72 |
| Swaziland | 2005 and 2006 | Evaluation of campaign to reduce multiple partners | National sample. 12 sites. 2,120 adults in 2005, 2,115 in 2006 | Fewer men in the follow-up reported concurrency and multiple partners than in the baseline. Men and women who had heard of the campaign were more likely to use condomsn with a non-regular partner.. In the follow-up, more women felt they could decide how many partners they had, and more men felt they were at risk of HIV. While the findings were compatible with an impact of the campaign, analysis of condom use in surveys before and after the campaign period indicated an overall decreasing rate of reporting multiple partners from 2002 to 2006, with some increase thereafter, especially among women. | 73 |

| Country | Year | Topic | Sample size and domain | Results, key findings | References |
|---|---|---|---|---|---|
| Uganda | 1995 | Agriculture and health services | 5,564 households, in 9 districts; 40 male and female focus group discussions | Some 38% of households rated government health services as good; 22% rated them as bad. Many complained about non-availability of drugs and poor access to health facilities. Around 25% of households had used government health services in the last month; 31% had used other health services. Two thirds of households were willing to pay for improved health facilities. 80% of recent service users reported drugs and treatment materials were available, and 20% reported they paid more than 1000 Ush for their visit. Focus groups complained about having to pay, even when service was poor, and about staff attitudes, especially towards poor people. | 74 |
| Uganda | 1998 | National integrity survey | 18,412 households, 1,595 service workers, 178 community key informants, 348 male and female focus group discussions | The social audit covered primary education and health, police, local administration, judiciary and revenue services. Only 27% of health service users knew how to make a complaint and only 12% had actually complained. Two thirds were satisfied with the service speed and staff behaviour. Some 28% had made an unofficial payment to service workers; those who paid took longer to complete their contact and were less satisfied with the service they received. Those who reported being given helpful information about using the services were less likely to have paid a bribe. Focus groups expressed anger and frustration about health services corruption. The findings informed the national integrity plan in Uganda. | 75 |
| West Bank and Gaza | 1998 | Health service delivery survey | 2,988 households, 42 primary care clinics; 51 secondary care facilities; 48 focus group discussions | A third of household heads were registered refugees. Half the households rated available health services as good. Most relied on secondary health facilities, because of easy access (especially after hours) and perceived better quality of service. More people rated their experience positively after a visit to an NGO/charity or private hospital (about 80%) than to a government or UNRWA (refugee services) hospital (about 65%). Service users made payments in all types of facilities; payment was more common and costs were higher in NGO/charity and private hospitals. A common complaint about both primary health facilities and hospitals was a perceived lack of medicines needed to treat common conditions. | 76 |
